# A Data-Driven Spatiotemporal Risk Assessment Framework for Transformer Overload in Distributed Renewable Energy System

**DOI:** 10.3390/s26113505

**Published:** 2026-06-02

**Authors:** Chengjun Xie, Chenhao Sun, Yanzheng Liu

**Affiliations:** State Key Laboratory of Disaster Prevention & Reduction for Power Grid, Changsha University of Science and Technology, Changsha 410114, China; cppcc95@gmail.com (C.X.); yanzheng_l@163.com (Y.L.)

**Keywords:** transformer overload risk assessment, edge-based static feature risk assessment, cloud-based dynamic feature risk assessment, cloud–edge parameter alignment and adjustment, risk score fusion, condition monitoring

## Abstract

In distributed renewable energy systems, load fluctuations caused by energy resources and energy storage increase the overload risk of distribution transformers, which may accelerate insulation aging and cause overheating, and undermine operational reliability. For transformer condition monitoring, this risk is reflected not by a single variable but by heterogeneous sensing observations acquired from electrical, thermal, and equipment status monitoring channels. Because full-scale inspection of latent defects is impractical under limited staffing and equipment resources, accurate overload risk prediction is important for sensor-driven maintenance allocation. With such motivations, this paper proposes a Transformer Overload Risk Assessment (TORA) approach for robust overload risk prediction under nonstationary load conditions. First, a feature matrix is constructed by jointly incorporating static features that capture long-term drift and dynamic features extracted from multisource sensing and supervisory signals that reflect short-term fluctuations. Then, static and dynamic features are assessed with Edge-based Static Feature Risk Assessment (E-SFRA) model and Cloud-based Dynamic Feature Risk Assessment (C-DFRA) model, respectively, according to their temporal and statistical characteristics. Next, a periodic calibration model (CE-PAA) is established through a cloud–edge loop, which uses low-latency edge updates and high-capacity cloud computation as feedback. Finally, risk score fusion (RSF) fuses generated static and dynamic risk scores to integrate cloud and edge strengths. The case study results indicate that TORA can transform heterogeneous monitoring signals into calibrated risk information in the studied single power plant scenario, providing useful support for multisource sensor data fusion, transformer condition monitoring, and maintenance decision making. Further validation using multi source field datasets is still needed to assess its cross scenario generalization ability.

## 1. Introduction

### 1.1. Motivation and Background

Amid intensifying global energy constraints and mounting environmental pressures, energy systems are shifting toward cleaner and more efficient paradigms, making sustainability a shared core objective worldwide. A central challenge of this transition is reconciling a secure energy supply with deep decarbonization. As the core nexus for energy conversion and delivery, the power system must reliably accommodate large-scale clean-energy integration and expanding end-use electrification to advance the dual-carbon goal. Among its assets, the distribution transformer, which links the generation, transmission, distribution, and end-user sides, plays an irreplaceable role: its operating condition directly shapes conversion efficiency and grid stability, underpinning secure, continuous supply, while a high-efficiency, low-carbon energy transition is also facilitated [1,2].

The loading condition of distribution transformers is a primary determinant of supply quality and reliability. With rising renewable-energy penetration and accelerating urbanization, both the frequency and magnitude of distribution-network peak demand continue to increase. Prolonged operation above rated capacity causes transformers to experience sustained stress, making them prone to heavy overloading [3]. Such sustained heavy loading not only jeopardizes the safety of the equipment itself but also triggers a cascade of secondary problems.

When a transformer remains under heavy loading for extended periods, the risk of equipment damage rises markedly. Specifically, heavy loading raises the operating temperature, especially the hot spot temperature. Once this exceeds its permissible limit, insulation ages and deteriorates more rapidly, which can ultimately lead to equipment failure [4]. Overheating or fires induced by heavy loading can cause transformer oil leakage, contaminating soil and water, disrupting ecological balance, and harming habitats and food chains, which run counter to the core green, low-carbon imperative of the energy transition [5]. In addition, when a transformer operates under heavy load, supply reliability is compromised: voltage instability and outages may occur, making it difficult to meet power quality and reliability requirements [6]. Constraints in staffing and equipment make continuous, system-wide inspection for latent defects impractical. Therefore, accurate prediction and assessment of transformer heavy-loading conditions are crucial to maintaining supply stability, advancing environmental sustainability, and efficient resource allocation [7].

This study targets day overload risk prediction for distribution transformers to support planned Operations and Maintenance (O&M). It does not address millisecond-to-second transient protection or fast tripping. From the perspective of sensor applications, the target problem is to convert heterogeneous monitoring observations, including electrical, thermal, and equipment-status information, into actionable overload-risk estimates for planned transformer condition monitoring and maintenance.

### 1.2. State of the Art and Limitations

For predicting and assessing heavy-overload conditions in distribution transformers, the literature has developed multiple technical pathways that can be grouped by technological generation into the following stages.

Early-stage assessment methods are grounded in physical laws and establish the relationship between load and equipment condition through analytical derivation. Their defining features include: taking the heat-balance equation and the law of electromagnetic induction as theoretical foundations; analytically deriving quantitative links between loading and condition; and accounting for multiphysics coupling effects as well as the computation of state parameters under different physical principles. For example, Yang et al. proposed a method based on dynamic mode decomposition (DMD) for transformer hot-spot temperature prediction, and it provides fast, accurate estimates of the hot-spot and winding temperature fields [8]. Blomgren et al. developed a transformer temperature-prediction model grounded in thermodynamic principles, addressing the online estimation and forecasting of hot-spot temperature in oil-immersed transformers [9]. Vahid Shiravand et al., building on a physical thermal model formulated as a nonlinear thermal-resistance network, introduce three additional thermal points at the radiator (top, middle, bottom) with a supporting estimation algorithm, thus improving the prediction accuracy of distribution transformers’ top-oil temperature and winding hot-spot temperature [10]. Yu et al. established a bidirectionally coupled electromagnetic–thermal–fluid numerical model for converter-transformer windings, overcoming the underestimation of hot-spot temperature caused by the traditional uniform-loss assumption, which jeopardizes transformer reliability [11]. Liu et al. presented an improved 1-D model that accounts for the horizontal proximity effect and non-uniform magnetic-field intensity, and by solving Maxwell’s equations and optimizing the winding structure, it corrects winding-loss calculation errors in high-insulation-voltage planar transformers [12]. These approaches have key limitations: reliance on analytical derivations limits their ability to capture real-world multiphysics couplings, and dependence on difficult-to-obtain physical parameters restricts assessment accuracy.

With advances in intelligent sensing, the limitations of physics-based models have catalyzed the rise of data-driven approaches, whose core is to learn the mapping from historical data to heavy-overload states. In practical deployment, these historical data are not abstract numerical records alone, but heterogeneous sensing streams collected from transformer monitoring and supervisory systems, such as electrical measurements, thermal observations, and operating-status signals. Therefore, transformer overload assessment is also a sensor-data interpretation and fusion problem under nonstationary operating conditions. In early studies on transformer overload prediction, shallow machine-learning methods such as support vector machines (SVM) and random forests were the mainstream choices [13]. Their key mechanism is manual feature engineering to extract salient indicators, followed by statistical learning to fit the relationship between features and heavy-overload status [14]. These methods offer notable advantages in interpretability, since model parameters often align with physical meaning, and in computational efficiency, which supports real-time assessment on commodity hardware [15]. However, they rest on assumptions that (i) fixed, hand-crafted features can adequately capture data regularities, and (ii) datasets are relatively small, well-structured, and stationary. In large-scale, noisy, and unstructured settings, shallow models struggle to capture deeper patterns and lack adaptive capacity, leading to marked declines in predictive accuracy and generalization [16]. At a finer level, different shallow models exhibit distinct weaknesses: SVM depends on manually curated feature sets, so suboptimal selection directly degrades diagnostic accuracy [17]; random forests, while capable of handling multi-feature inputs, show generalization sensitivity to the underlying data distribution, are susceptible to noise, and are affected by class imbalance [18].

To overcome the feature-engineering bottleneck of shallow learning, deep learning methods have been increasingly applied to transformer fault assessment. In heavy-overload prediction, deep models have become a research focus owing to their strong nonlinear fitting capacity, with convolutional neural networks (CNNs) and recurrent neural networks (RNNs), including LSTM/GRU variants, serving as mainstream choices [19]. Their core mechanism is to automatically extract spatiotemporal features from load data via multi-layer networks: CNNs leverage convolutions to capture local salient patterns and detect abrupt load anomalies [20], while RNNs and their variants specialize in long- and short-term sequence dependencies, uncovering seasonal and diurnal periodicities in transformer loading [21]. These approaches obviate manual feature construction and can learn complex mappings directly from raw inputs (historical load, temperature, weather, etc.), and when data are abundant, they often deliver high predictive accuracy [22]. That said, their effectiveness presupposes large-scale, high-quality labeled data, relatively stable data distributions, and lower interpretability requirements. In small-sample regimes, under distributional dynamics, or in settings that require real-time explanation, limited adaptability can lead to pronounced drops in accuracy and reliability [23]. More specifically, CNNs are data-hungry, computationally expensive, and training-intensive, and they are prone to overfitting and may suffer from loss of spatial information and limited robustness to complex transformations [19]. RNNs/LSTM/GRUs experience memory decay when modeling long-horizon dependencies associated with long-cycle faults and are sensitive to high-noise monitoring data [24]. Moreover, the black-box nature of deep models weakens interpretability, making it difficult for O&M personnel to understand the basis of “heavy-overload risk” decisions and to trust the outputs in real dispatch practice [25].

Recent studies have further extended deep learning-based fault diagnosis and risk assessment from single-domain sequence modeling to transfer learning, graph interaction, and fault-tolerant decision support. For example, conditional distribution-guided adversarial transfer learning has been developed to reduce both marginal and conditional distribution discrepancies between multiple source domains and a target domain, improving cross-domain diagnostic adaptability under cross-machine and distribution-shift scenarios [26]. Spatial-channel collaborative multi-scale graph interaction deep transfer learning has been proposed to refine multi-domain feature prototypes in both spatial and channel dimensions and to construct a multi-scale graph interaction network for deeper cross-domain feature interaction, which is useful for extracting transferable fault representations under unlabeled target-domain and distribution-shift scenarios [27]. In addition, recent fixed-time adaptive fault-tolerant control studies have shown that adaptive control mechanisms can improve system robustness under actuator faults, random disturbances, model uncertainties, and mode-transition conditions [28]. These studies indicate that transfer learning, graph interaction, and fault-tolerant mechanisms can improve robustness in intelligent diagnosis and decision support. However, most focus on cross-domain machinery fault diagnosis or post-fault control compensation, while the current transformer overload problem emphasizes day-to-multi-day risk prediction from heterogeneous monitoring signals, static–dynamic feature separation, cloud–edge parameter alignment, and calibrated risk fusion for planned O&M. Therefore, TORA complements existing deep transfer learning and fault-tolerant control studies by focusing on sensor-driven overload-risk assessment and maintenance prioritization in distributed renewable energy systems.

In summary, despite multiple rounds of progress from physics-based modeling to shallow learning and to deep learning, current research on transformer-heavy overload prediction still faces two key gaps:

1. Lack of unified static–dynamic modeling and adaptive fusion: Static and dynamic features are often processed in a single stream, ignoring their markedly different statistical properties and temporal variability, which blurs risk semantics, undermines comparability and calibration consistency, and hampers an interpretable, unified risk baseline. In practice, even when the static–dynamic distinction is acknowledged, fusion typically relies on fixed linear weights for fusion without adapting to operating conditions or uncertainty, which in turn causes instability under nonstationarity and reduces early-hit efficiency and ranking robustness.
2. Lack of cloud–edge integration and field-center alignment: many deployments are centralized or edge-isolated, the edge’s network conditions are misaligned with the center’s training/update cadence, and the lack of integrated coordination and versioned governance prevents a closed loop between model updating and online inference, delaying responses to scenario and distribution changes.

### 1.3. Research Statements and Contributions

To remedy the deficiencies in transformer heavy-overload prediction, this paper proposes TORA for day-to-multi-day planned O&M, providing a coordinated solution across data organization, static modeling, dynamic modeling, cloud–edge orchestration, and fusion evaluation. From a sensing-system perspective, TORA provides a deployable framework for integrating multisource monitoring signals, handling their distributional differences across cloud and edge, and transforming them into calibrated risk information for condition monitoring and maintenance decision support. First, a more comprehensive feature-variable matrix is constructed by jointly incorporating static features capturing long-term drift and dynamic features reflecting short-term fluctuations, with broader feature diversity and coverage across types and data modalities. Second, on the edge, the Edge-based Static Feature Risk Assessment (E-SFRA) model learns prototype structures of the static profile and together with monotonic calibration, outputs interpretable, low-latency static scores, thus reducing reliance on hard-to-measure mechanistic parameters and improving generalizability; in the cloud, the Cloud-based Dynamic Feature Risk Assessment (C-DFRA) model captures multivariate long-term and short-term dependencies and interactions to produce a dynamic score, enhancing robustness and interpretability under complex time series and un/semi-structured data. Third, Cloud–Edge Parameter Alignment and Adjustment (CE-PAA) periodic calibration builds a cloud–edge feedback loop by delivering signed, versioned calibration packages to the edge on a periodic or event-driven basis, and after validation, the system updates static-branch parameters via EMA, preserves historical scores, and maintains full logging, audit, and rollback capabilities. Finally, risk score fusion (RSF) fuses the edge static and cloud dynamic risk scores with an uncertainty-aware softmax-gated mixture-of-experts, deriving weights from branch uncertainties and cloud quality, producing a unified risk index.

The contributions of this paper are outlined as follows:

- Multi-feature incorporation: Static features address long-term drifts, while dynamic features address short-term fluctuations. Given their distinct distributional characteristics, both two types are analyzed by E-SFRA and C-DFRA, respectively, to thus overcome the potential inherent time-scale mismatch between them.
- Cloud–edge periodic calibration mechanism: CE-PAA model is formed to periodically retune model calibration parameters within a cloud–edge loop, where the edge is deployed for low-latency updates and the cloud for high-capacity computation, thus combining as a cloud–edge feedback channel.
- Adaptive fusion mechanism: Both static and dynamic risk scores are adaptively fused by uncertainty-aware weighting according to the branch uncertainties in the initiated RSF ensemble, and therefore cooperatively integrating the strengths of cloud and edge.
- Sensor-oriented application value: The proposed framework integrates heterogeneous monitoring signals from transformer sensing and supervisory systems, supports low-latency edge-side warning together with cloud-side analysis, and provides calibrated overload-risk information for practical condition monitoring and maintenance decision support.

The remainder of this paper is organized as follows: Section 2 constructs the feature matrix; Section 3 develops the TORA framework, including preprocessing, E-SFRA, C-DFRA, CE-PAA, and RSF; Section 4 presents the end-to-end workflow; Section 5 reports an empirical case on heavy-overload prediction for distribution transformers; Section 6 concludes with the main findings of the research.

## 2. Data Preparation and Feature Engineering

No additional experimental equipment, materials, or measuring devices were newly deployed in this study.Data processing, model development, and performance evaluation were conducted using MATLAB R2024a and Python 3.12.

### 2.1. Construction of the Static–Dynamic Feature System and Selection Criteria

In this study, features are partitioned by their dependence on time and operating conditions (not by fluctuation magnitude) into static and dynamic sets. Static features support routine risk identification and planned O&M: they are long-term stable, comprising (i) intrinsic attributes fixed at factory/installation and independent of operating state, and (ii) attributes that may change but on cycles far longer than the 1 h~7 days assessment horizon, so they are treated as constant for short-term analysis. Owing to this stability, static features continuously reflect baseline risk and underpin periodic maintenance planning. Dynamic features capture short-term operating state: they vary with load, loading rate, and temperature, are sampled at ≤1 h intervals, and exhibit fine-grained temporal structure. Their value is less about latency than about accuracy and temporal dependence. Consequently, dynamic features delineate near-term stress/risk boundaries and support accurate short-term overload prediction. Most of these dynamic features are sensor-derived monitoring variables acquired from transformer sensing and supervisory systems, including electrical measurements, thermal observations, and equipment-status signals.

Based on the above feature-partitioning principle, the selected variables are divided into static and dynamic features, as summarized in Table 1 and Table 2 respectively.

Table 2 mainly consists of sensor-derived monitoring variables collected from transformer sensing and supervisory systems, covering electrical measurements, thermal observations, and equipment-status signals for practical condition monitoring.

### 2.2. Construction of the Feature-Database Matrix

Let $L=\left\{l_{1},l_{2},\ldots ,l_{i},\ldots ,l_{m}\right\}$ denote the label set corresponding to the fault records. Let $B=\left\{b_{1},\ldots ,b_{j},b_{j+1},\ldots ,b_{n}\right\}$ denote the set of input features related to transformer heavy-overload, where $b_{j}$ is a single input feature variable. Each input feature $b_{j}$ is composed of a set of feature elements $\left\{c_{1,j},\ldots ,c_{i,j},\ldots ,c_{m,j}\right\}$. Let $G=\left\{g_{1},g_{2},\ldots ,g_{i},\ldots ,g_{m}\right\}$ denote the set of m target variables, where $g_{i}$ represents the transformer’s heavy-overload state. Let $E=\left\{e_{1},e_{2},\ldots ,e_{i},\ldots ,e_{m}\right\}$ be the set of transformer identifiers, where $e_{i}$ is the ID of the transformer associated with each daily fault record. Let $T=\left\{t_{1},t_{2},\ldots ,t_{i},\ldots ,t_{m}\right\}$ denote the set of timestamps, where $t_{i}$ is the specific time of the fault. The constructed matrix consists of five components: a label vector, an input-feature matrix, a target vector, a transformer-ID vector, and a time vector. The first row of the matrix corresponds to the data headers. Based on these definitions, the processing-space matrix DM for transformer heavy-overload status prediction is constructed as follows:

(1) $${L=\left\{l_{1},l_{2},\ldots ,l_{i},\ldots ,l_{m}\right\}}^{T}$$

(2) $$B=\left[\begin{array}{cc} \begin{array}{ccc} b_{1} & \ldots & b_{j} \end{array} & \begin{array}{ccc} b_{j+1} & \ldots & b_{n} \end{array}\\ \begin{array}{ccc} \begin{array}{c} c_{1,1}\\ \vdots \\ \begin{array}{c} c_{i,1}\\ \vdots \\ c_{m,1} \end{array} \end{array} & \begin{array}{c} \ldots \\ \ddots \\ \begin{array}{c} \ldots \\ \ddots \\ \ldots \end{array} \end{array} & \begin{array}{c} c_{1,j}\\ \vdots \\ \begin{array}{c} c_{i,j}\\ \vdots \\ c_{m,j} \end{array} \end{array} \end{array} & \begin{array}{ccc} \begin{array}{c} c_{1,j+1}\\ \vdots \\ \begin{array}{c} c_{i,j+1}\\ \vdots \\ c_{m,j+1} \end{array} \end{array} & \begin{array}{c} \ldots \\ \ddots \\ \begin{array}{c} \ldots \\ \ddots \\ \ldots \end{array} \end{array} & \begin{array}{c} c_{1,n}\\ \vdots \\ \begin{array}{c} c_{i,n}\\ \vdots \\ c_{m,n} \end{array} \end{array} \end{array} \end{array}\right]$$

(3) $${G=\left\{g_{1},g_{2},\ldots ,g_{i},\ldots ,g_{m}\right\}}^{T}$$

(4) $${E=\left\{e_{1},e_{2},\ldots ,e_{i},\ldots ,e_{m}\right\}}^{T}$$

(5) $$T={\left\{t_{1},t_{2},\ldots ,t_{i},\ldots ,t_{m}\right\}}^{T}$$

(6) $$DM=\left\{L\;B\;G\;E\;T\right\}$$

In this formulation, each element $l_{1},l_{2},\ldots ,{l_{i},\ldots ,l}_{m}\;$in the label vector L denotes the fault label corresponding to a specific fault record. In the input-feature matrix B, the first row $b_{1},\ldots ,b_{j},b_{j+1},\ldots ,b_{n}$ lists the names of input features related to transformer heavy-overload. Specifically, columns $b_{1}$ through $b_{j}$ correspond to static features, whereas columns $b_{j+1}$ through $b_{n}$ correspond to dynamic features. For each fault record, the feature variables are recorded starting from the second row. Here, $c_{i,j}$ is an element of the input-feature matrix B, representing the feature factor for the fault record with label $l_{1}$, feature $b_{j}$, transformer ID $e_{i}$, and timestamp $t_{i}$. The combination of multiple features jointly determines the corresponding target variable $g_{i}$ in the target vector G.

## 3. Establishment of the TORA Model

### 3.1. Edge-Based Static Feature Risk Assessment (E-SFRA)

On the edge, an improved LVQ [29] compresses static features into a device-level risk score $R_{S}$ under tight latency, providing a fast, low-cost reference for routine O&M.

#### 3.1.1. Static Feature Preprocessing

This section targets static risk-degree modeling for the equipment’s inherent attributes. Let the preprocessed vector of the *i*-th transformer (sample) over the static feature set be $x_{i}=\left[c_{i,1},\ldots ,c_{i,j}\right]$, where numerical features are Min–Max normalized to [0, 1], categorical features are converted into binary vectors via one-hot encoding, and missing/highly correlated features have been removed according to standard procedures.

#### 3.1.2. The E-SFRA Model

**Figure 1:**
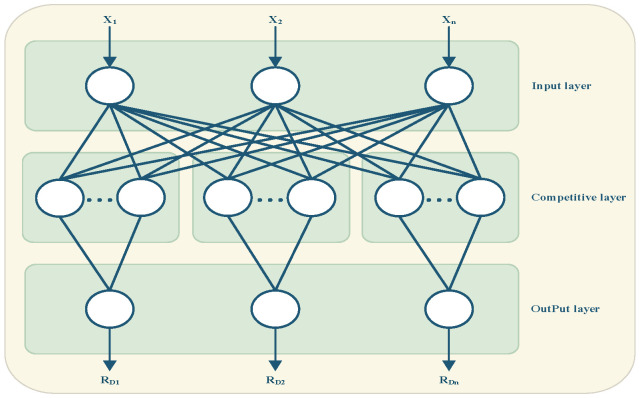
LVQ network architecture.

After completing data preprocessing and constructing the feature database matrix DM, for the static feature columns from $b_{1}$ to $b_{j}$, this paper adopts the E-SFRA model based on the improved LVQ to perform risk scoring, thus obtaining the comprehensive static risk $R_{S}$. The LVQ neural network is a feedforward supervised neural network with a trained competitive layer, consisting of three layers of neurons: an input layer, a competitive layer, and an output layer. Figure 1 shows the LVQ network architecture.

The input layer serves as the data conduit, feeding $x_{i}$ into the competitive layer, where the winner-takes-all (WTA) learning mechanism selects the prototype that best matches the sample and computes the weighted distance.

The competitive layer consists of K prototype vectors (prototype vectors), denoted as $\left\{\omega_{k,1},\omega_{k,2},\ldots ,\omega_{k,j}\right\}$, each prototype representing a typical static risk pattern. To characterize the importance of different static features for risk discrimination, a global correlation weight $\lambda =\left\{\lambda_{1},\lambda_{2},\ldots ,\lambda_{j}\right\}$ satisfying $\lambda_{j}\geq 0$ and $\sum_{j}\lambda_{j}=1$. The weighted squared Euclidean distance is used to compute the distance between the input vector $x_{i}$ and the K-th prototype vector $\omega_{k}$:

(7) $$d_{k}\left(x_{i}\right)=\sum_{j=1}^{j}\lambda_{j}{\left(c_{i,j}-\omega_{k,j}\right)}^{2}$$

The input sample is assigned to the “winning prototype” with the smallest distance, thus determining its risk pattern.

We adopt a prototype discriminative learning scheme based on the weighted squared Euclidean distance. For any sample $\left(x,y\right)$, denote the nearest same-class/different-class prototype distances as: $\omega^{+}=arg\underset{k:c\left(\omega_{k}\right)=y}{\min}d_{k}\left(x_{i}\right)$, $\omega^{-}=arg\underset{k:c\left(\omega_{k}\right)\neq y}{\min}d_{k}\left(x_{i}\right)$ and let $d^{+}=d_{\omega^{+}}\left(x_{i}\right)$, $d^{-}=d_{\omega^{-}}\left(x_{i}\right)$. Use the hinge-style margin loss:

(8) $$L\left(x_{i}\right)=\;\alpha_{y}max\left(0,{d^{+}-d}^{-}+\delta \right),\delta >0$$

Jointly update prototypes and correlation weights via a first-order method (project λ onto the simplex at each step); initialize with class means/K-means; apply early stopping based on validation-set metrics.

Once the competitive layer has determined the sample’s distances to all prototypes, the output layer converts the distances into softmin similarities and performs an all-prototype fusion to obtain the uncalibrated risk $s_{k}\left(x_{i}\right)$:

(9) $$s_{k}\left(x_{i}\right)=\frac{exp\left(-\beta d_{k}\left(x_{i}\right)\right)}{\sum_{\ell =1}^{K}exp\left(-\beta d_{\ell }\left(x_{i}\right)\right)}\;\;\;\;\;\;\left(\beta >0\right)$$

(10) $$R_{raw}\left(x_{i}\right)=\sum_{k=1}^{K}s_{k}\left(x_{i}\right){\cdot r}_{k}$$

where β is the temperature coefficient with β>0. $r_{k}$ is the (responsibility-weighted) empirical mean of labels for samples covered by prototype k, clipped to [0,1]. To ensure that the output has probabilistic meaning and falls within the standardized interval, isotonic regression is further applied for monotonic fitting to obtain the final static risk score:

(11) $$R_{S}\left(x_{i}\right)=g\left(R_{raw}\left(x_{i}\right)\right)\in \left[0,1\right]$$

Consequently, the model outputs a continuous 0–1 static-feature risk score that intuitively reflects the overload risk level of a transformer at the inherent-attribute level and this score can be used directly for edge-side risk monitoring and early warning.

### 3.2. Cloud-Based Dynamic Feature Risk Assessment (C-DFRA)

**Figure 2:**
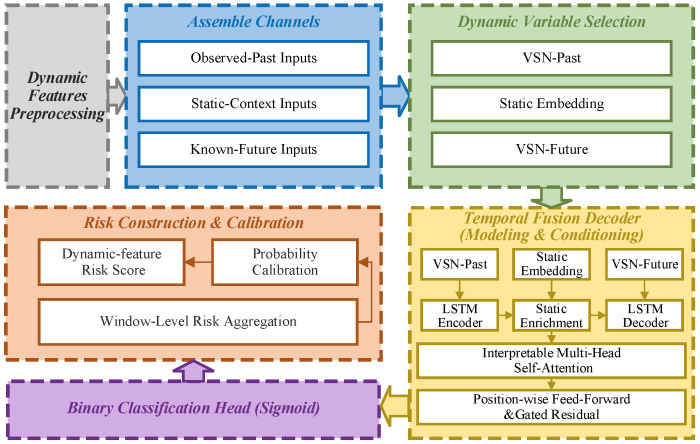
C-DFRA model structural schematic.

Dynamic features encode equipment state and temporal variation, requiring high-accuracy models to capture nonlinear and latent relations. Leveraging cloud compute, we deploy complex deep models to model these features and assess risk, producing a comprehensive dynamic-feature risk $R_{D}$. The cloud setting ensures accuracy and model depth, and short-term trend forecasting and risk-boundary identification are achieved. Accordingly, we build a cloud-side time-series pipeline based on TFT [30], using historical observations and known future covariates as inputs and outputting $R_{D}$. Figure 2 shows the C-DFRA architecture.

#### 3.2.1. Dynamic Feature Preprocessing

From the feature database DM constructed in Section 2.2, this paper extracts the dynamic subset from the input matrix B: $B_{\mathrm{dynamic}}=\{b_{j+1},\ldots ,b_{n}\}$, with elements $\left\{c_{i,j+1},\ldots ,c_{i,n}\right\}$ (i indexes fault records). Raw dynamic data are standardized: align timestamps/time zones and unify sampling interval; aggregate load/current by window maxima (to preserve limit-exceeding peaks), temperature/humidity by window means, and cumulative quantities by window sums. Samples are then built via sliding windows and assembled to match TFT’s three-stream inputs: historical observations, known future covariates, and static context.

#### 3.2.2. The C-DFRA Model

TFT takes the standardized sequence as input and outputs the binary probabilities for the next H steps; the window-level risk—i.e., the dynamic-feature risk score $R_{D}$ is then constructed in post-processing. We adopt the TFT pipeline “channel assembly → variable selection (VSN) → LSTM encoder–decoder → interpretable multi-head self-attention → binary classification,” with window-level risk aggregation and probability calibration performed in post-processing.

Let the time index be t=1,2,…,T and the forecasting horizon h=1,2,…,H. The inputs are split into historical observations $x_{t}^{\mathrm{past}}\in R^{d_{p}}$, known-future covariates $x_{t}^{\mathrm{kf}}\in R^{d_{f}}$, and static covariates $s\in R^{d_{s}}$ (embedding the static risk from the previous section as $s=Embed(R_{S})$). The prediction target is a binary label $g_{t+h}\in \{0,1\}$ indicating whether an overload occurs at step h ahead. To ensure subsequent residual connections, all variables are first embedded/linearly projected to a common dimension $d_{\mathrm{model}}$.

To score and project variables on the past and future paths separately, we use a conditional Gated Residual Network (GRN). Define

(12) $$GRN\left(u;c\right)=LayerNorm\left(u+GLU\left(W_{2}ELU(W_{1}u+W_{c}c)+b\right)\right)$$

(13) $$GLU\left(a\right)=\sigma \left(W_{g}a+b_{g}\right)⊙a$$

For the past branch, we first compute an importance score for each variable at time t and then normalize the scores:

(14) $$e_{k,t}^{past}=GRN\left(\left[x_{t}^{past};s\right];s\right)$$

(15) $$\alpha_{k,t}^{past}=\frac{exp(e_{k,t}^{past})}{\sum_{k^{\prime }}exp(e_{k^{\prime },t}^{past})}$$

Subsequently, we aggregate them using the weights and apply a conditional projection to obtain the selected representation:

(16) $${\tilde{x}}_{t}^{past}=\sum_{k}\alpha_{k,t}^{past}GRN\left(x_{k,t}^{past};s\right)$$

Analogously, the future branch produces ${\tilde{x}}_{t}^{kf}$; the static branch contains only the scalar $R_{S}$, so context vectors are provided directly by the static encoder for conditioning, and no separate static VSN is constructed.

The past/future streams are then fed into an LSTM encoder–decoder to obtain ϕ(t,n), with the decoder initialized by $\left(c_{c},c_{h}\right)$; subsequently, a gated residual alignment is applied for stabilization:

(17) $$\tilde{\phi }\left(t,n\right)=LayerNorm\left({\tilde{x}}_{t+n}+{GLU}_{\phi }\left(\phi \left(t,n\right)\right)\right)$$

Condition the temporal features with the static context:

(18) $$\theta \left(t,n\right)={GRN}_{\phi }\left(\tilde{\phi }\left(t,n\right),c_{e}\right)$$

Apply scaled dot-product self-attention to Θ(t)={θ(t,n)} with shared $W_{V}$ across heads and head-wise averaging, and then perform a gated residual fusion:

(19) $$A=softmax\left(\frac{\Theta W_{Q}{\left(\Theta W_{K}\right)}^{T}}{\sqrt{d}}+M_{causal}\right)$$

(20) $$\beta =A\left(\Theta W_{V}\right)$$

(21) $$\delta \left(t,n\right)=LayerNorm\left(\theta \left(t,n\right)+{GLU}_{\delta }\left(\beta \left(t,n\right)\right)\right)$$

After the position-wise feed-forward layer, perform a gated residual fusion with $\tilde{\phi }$, producing the representation used by the task head:

(22) $$\psi \left(t,n\right)={GRN}_{\psi }\left(\delta \left(t,n\right)\right)$$

(23) $$\tilde{\psi }\left(t,n\right)=LayerNorm\left(\tilde{\phi }\left(t,n\right)+{GLU}_{\psi }\left(\psi \left(t,n\right)\right)\right)$$

Set the decoder representation $z_{h}$ to $\tilde{\psi }(t,h)$.

For each forecast step h, a linear classification head followed by a sigmoid produces the probability of an overload at time t+h:

(24) $$l_{t+h}=W^{\left(out\right)}z_{h}+b^{\left(out\right)}$$

(25) $$p_{t+h}=\sigma \left(l_{t+h}\right)$$

Training objective (binary). We use weighted binary cross-entropy to address class imbalance:

(26) $$L=-\sum_{h=1}^{H}\left[\omega_{1}g_{t+h}\log(p_{t+h})+\omega_{0}(1-g_{t+h})\log(1-p_{t+h})\right]$$

where $\omega_{1}$ and $\omega_{0}$ are the positive- and negative-class weights, respectively. To improve reproducibility and avoid information leakage, the class weights were calculated only from the training set using the inverse-frequency strategy${\;\omega }_{1}=N/(2N_{1})$, ${\;\omega }_{0}=N/(2N_{0})$, where $N_{1}$ and $N_{0}$ denote the numbers of positive and negative training samples, respectively, and $N\;={\;N}_{1}\;+\;N_{0}$. In this study, the training set contains 430 positive samples and 4930 negative samples. Therefore, ${\;\omega }_{1}=6.233$ and ${\;\omega }_{0}=0.544$.

The binary label $g_{t+h}$ was defined according to the transformer loading rate and thermal operating condition. The primary heavy-overload criterion is defined as the transformer loading rate exceeding 120% for at least 15 consecutive minutes, corresponding to three consecutive 5 min sampling intervals. In addition, to reflect thermal stress and equipment-condition effects, samples with a loading rate above 115% were also labeled as heavy-overload-related high-risk samples when accompanied by high winding temperature, high top-oil temperature, rapid temperature rise, cooling limitation, or high static baseline risk. Otherwise, the sample was labeled as non-heavy-overload.

Unlike the original TFT, which uses a quantile output head with the pinball loss, this work replaces the output head with a binary probability head and adopts weighted binary cross-entropy (BCE) to accommodate the overloaded labels and class imbalance.

The model directly outputs the stepwise exceedance probabilities $\{p_{t+h}{\}}_{h=1}^{H}$. We then aggregate them into a window-level risk under the criterion “at least one exceedance within the future window”:

(27) $${\hat{R}}_{D}\left(t\right)=1-\prod_{h=1}^{H}\left(1-p_{t+h}\right)$$

On the validation set, perform probability calibration to obtain:

(28) $$R_{D}=Calibrate\left({\hat{R}}_{D}\left(t\right)\right)\in \left[0,1\right]$$

### 3.3. Cloud–Edge Parameter Alignment and Adjustment (CE-PAA)

**Figure 3:**
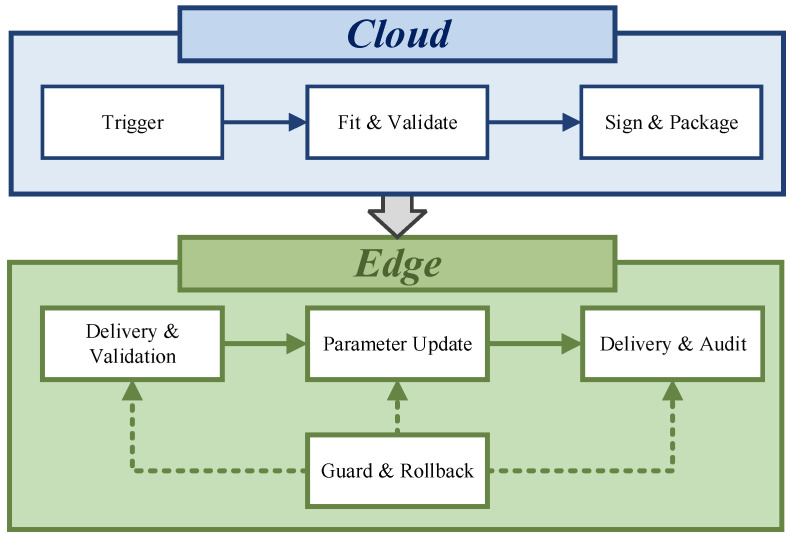
Architecture of CE-PAA.

This paper next details the Cloud–Edge Parameter Alignment and Adjustment (CE-PAA) workflow (Figure 3). Figure 3 shows the structure of this mechanism.

CE-PAA updates only the parameter layer of the static branch defined in Section 3.1, with updates taking effect before the next forward inference; it does not overwrite any previously released static risk scores. The structural components remain frozen. This design establishes a stable cloud–edge feedback.

loop: the cloud learns improved parameters, and the edge applies them prospectively.

Triggering and packaging. As shown in Figure 3, CE-PAA is triggered on a periodic schedule (daily/weekly/monthly) and by drift or policy events. The cloud fits and validates a parameterized calibration head on a holdout set, and assembles a signed parameter package.

(29) $$\Pi =\left\{T,\gamma ,\left(\mu ,\sigma \right),ver,TTL,sig,q_{d}\right\}$$

where T is the temperature factor for the static-branch logits; γ parameterizes the monotonic calibrator and satisfies a non-decreasing constraint; (μ,σ) are the feature-wise normalization statistics; and $q_{d}$ is the quality score.

Delivery & validation. Upon receipt, the edge verifies the signature, checks version monotonicity and TTL freshness, and filters according to $q_{d}$. If any item fails, the package is rejected and the previous version is retained, while logging “missing/expired/low-quality” events; only approved packages proceed to parameter updating.

Parameter update. Let the forward pass of the static branch be

(30) $$z=g\left(x;\theta_{e}\right)$$

(31) $$p_{pre}=\sigma \left(\frac{z}{T_{e}}\right)$$

(32) $$\;\;R_{s}={Iso}_{\gamma }\left(p_{pre}\right)$$

where $\theta_{e}$ denotes the frozen structural weights from Section 3.1; $T_{e}$ and γ belong to the parameter layer; σ(⋅) is the Sigmoid function; and ${Iso}_{\gamma }(\cdot )$ is a non-decreasing monotonic calibrator.

CE-PAA performs soft updates on the parameter layer via an exponential moving average (EMA):

(33) $$T_{e}^{\left(t+1\right)}=(1-\rho )\;T_{e}^{\left(t\right)}+\rho \;T$$

(34) $$\gamma^{\left(t+1\right)}=(1-\rho )\;\gamma^{\left(t\right)}+\rho \;\gamma_{\mathrm{cloud}}$$

(35) $$\mu_{j}^{\left(t+1\right)}=(1-\rho )\;\mu_{j}^{\left(t\right)}+\rho \;\mu_{j,\mathrm{cloud}},\sigma_{j}^{\left(t+1\right)}=(1-\rho )\;\sigma_{j}^{\left(t\right)}+\rho \;\sigma_{j,\mathrm{cloud}}$$

where ρ∈(0,1) controls the update strength. This section does not rewrite any previously released $R_{s}^{\left(t\right)}$ and updates take effect only for subsequent inference and produce a new $R_{s}^{\left(t+1\right)}$ under the new version.

Delivery & audit. Forward $\left\{R_{s}^{\left(t+1\right)},\;R_{D},\;ver,\;TTL,\;q_{d}\right\}$ to the fusion module (Section 3.4). Log, by version and time, ECE, Brier, grade-level FPR/TPR, package acceptance/rollback events, and latency for governance and traceability.

Guard & rollback. If the package fails validation, is expired, or is low quality, or if reliability degradation is observed in the subsequent time window, roll back to the previous Π and keep the existing caliber.

### 3.4. Risk Score Fusion (RSF)

Following the parameter-only updates in Section 3.3, the edge LVQ outputs $R_{S}$ and the cloud TFT outputs $R_{D}$ (with quality $q_{d}$ and TTL). RSF is an uncertainty-aware [31] softmax-gated mixture-of-experts (UAG) [32] that derives weights from branch uncertainties and cloud freshness/quality, and adds a second-order interaction to model static–dynamic synergy, producing the unified risk index R. Let $u_{s}$ and $u_{d}$ denote the branch uncertainties and define the cloud freshness $\phi_{d}$ from $q_{d}$ and TTL; construct confidences $c_{s},c_{d}$ and obtain nonnegative, sum-to-one weights $\omega_{s},\omega_{d}$ via softmax, then fuse $R_{S}$ and $R_{D}$ to obtain R.

In the fusion stage, the quantities used are as follows: $R_{S}$ and $R_{D}$ both lie in [0, 1]; uncertainties $u_{s},u_{d}\in \left[0,1\right]$; freshness $\phi_{d}\in \left[0,1\right]$; confidences $c_{s},c_{d}\in \left[0,1\right]$; weights $\omega_{s},\omega_{d}\in \left[0,1\right]$ with $\omega_{s}+\omega_{d}=1$. θ denotes the exceedance threshold, H the prediction horizon, and $clip\left(x,0,1\right)$ denotes interval truncation.

The dynamic-branch uncertainty [33] is quantified via predictive entropy:

(36) $$u_{d}=-\frac{\left[R_{D}\log R_{D}+\left(1-R_{D}\right)\log\left(1-R_{D}\right)\right]}{\log2}$$

The static-branch uncertainty is approximated by the normalized Brier score:

(37) $$u_{s}=clip\left(\frac{1}{0.25}\frac{1}{N}\sum_{i=1}^{N}{\left(y_{i}-R_{S,i}\right)}^{2},0,1\right)u_{d}$$

Considering the freshness and quality of the cloud package, define $\phi_{d}$ as:

(38) $$\phi_{d}=\left\{\begin{array}{c} q_{d},unexpired\\ \beta_{0}q_{d},expired \end{array}\right.,\;\;\;0\leq \beta_{0}\leq 1$$

According to the principle that “lower uncertainty and higher quality imply greater reliability,” construct the branch confidences and obtain the weights via softmax.

(39) $$c_{s}=1-u_{s}$$

(40) $$c_{d}=\phi \left(1-u_{d}\right)$$

(41) $$\left(\omega_{s},\omega_{d}\right)=softmax\left(c_{s},c_{d}\right)$$

Finally, the comprehensive risk index R is defined as:

(42) $$R=\omega_{s}\cdot R_{S}+\omega_{d}\cdot R_{D}+\beta \cdot R_{S}{\cdot R}_{D}\;\;\;(\beta \geq 0)$$

The interaction term $\beta \cdot R_{S}{\cdot R}_{D}$ is used to characterize the potential synergistic effect when both static and dynamic risks are high. When the static risk and dynamic risk are simultaneously at elevated levels, this term amplifies the comprehensive risk value, reflecting the fact that a transformer is more prone to failure under high baseline risk and high operational stress.

## 4. The Operation Procedure of the TORA Model

By applying the above methods, this study establishes the Transformer Overload Risk Assessment (TORA) to predict the overload status of the transformer. The specific implementation process is as follows:

1. Static/Dynamic feature partitioning and selection: Obtain the input static and dynamic features according to the classification and screening criteria for static and dynamic features.
2. Construction of the feature database matrix: Organize labels L, input features B, target variables G, transformer identifiers E, and time vector T to form a processed spatial matrix for overload prediction.
3. Static feature preprocessing: Apply Min–Max normalization to numerical features; apply one-hot encoding to categorical features, outputting a clean static input vector for edge-side modeling.
4. Edge-side static risk modeling: On the edge, use the E-SFRA model to assess static features one by one and obtain the static risk value $R_{S}$, balancing real-time performance and low computational cost.
5. Iterative computation over static features: Repeat Step 4 for each static feature in the database to compute the comprehensive static risk value $R_{S}$.
6. Dynamic feature preprocessing: First, align time zones and timestamps according to the task step Δ and perform peak-preserving resampling; then mark missing/outliers and generate masks via leakage-free, training-window-only minimal imputation; finally, use sliding-window slicing to construct “history-known future-target” samples, outputting aligned sequences and masks for C-DFRA assembly.
7. Cloud-side dynamic risk modeling: Based on standardized dynamic features, deploy C-DFRA on the cloud (including LSTM for short-term dependencies, Transformer positional-encoding for long-term dependencies, VSN for variable selection, etc.) to output the dynamic risk score $R_{D}$.
8. Iterative execution over dynamic features: Repeat Step 7 for each dynamic feature in the database to compute the comprehensive dynamic risk value $R_{D}$.
9. Cloud–edge parameter refresh (CE-PAA): The cloud, periodically or on drift, dispatches validated calibration/gating parameters; the edge updates the parameter layer only, taking effect for subsequent inference without overwriting past risk values.
10. Risk score fusion (with uncertainty estimation): Introduce gating weights $\left[\omega_{s},\omega_{d}\right]$ driven by uncertainty and scenario characteristics; combine monotonic calibration and the static–dynamic interaction term to fuse $R_{S}$ and $R_{D}$ into the comprehensive risk index R.
11. Performance validation: Finally, normalize the comprehensive risk index R to 0–1 (0 → 1: from “impossible” to “certain”), and compare it with actual overload records (0 or 1: occurred or not) in the test set to validate the performance of the proposed prediction model.

**Figure 4:**
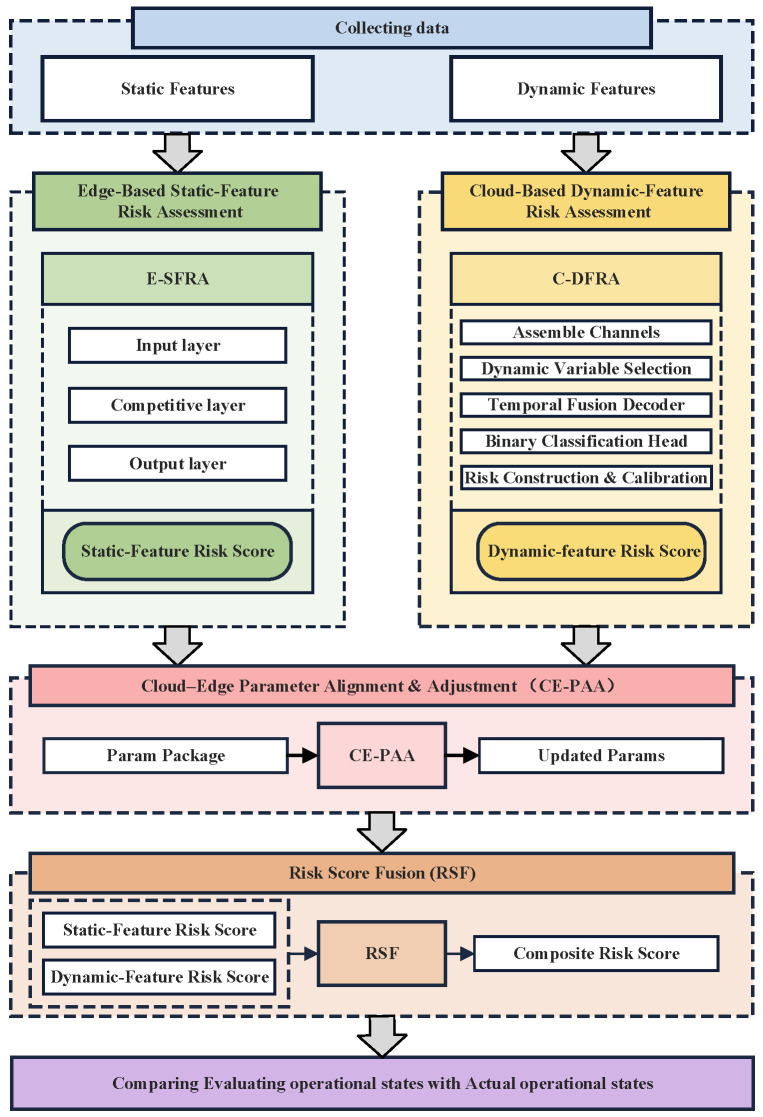
Flowchart of TORA.

Based on the above discussion, we construct a transformer overload prediction model using TORA. The basic flowchart is shown in Figure 4.

## 5. Empirical Case Study

### 5.1. Test Data

The experimental data used in this study come from the operating records of distribution transformers at a power plant in a province of China, encompassing two categories: inherent equipment attributes and operational monitoring data, with a total of 6700 samples. The dataset includes static features (e.g., rated capacity, model type, years in service, cooling method) and dynamic features (e.g., load factor, load growth rate, oil temperature, ambient temperature), jointly providing a comprehensive characterization of each transformer’s inherent carrying capacity and operating stress. The dynamic variables are sensor-derived monitoring quantities collected from transformer sensing and supervisory systems, mainly covering electrical measurements, thermal observations, and equipment-status signals for practical condition monitoring. To ensure methodological rigor and prevent information leakage, the data were strictly partitioned in chronological order into training, validation, and test sets in an 8:1:1 ratio.

Before model training, the heavy-overload label was defined according to the transformer loading rate and thermal operating state. The primary heavy-overload criterion was that the loading rate exceeded 120% for at least 15 consecutive minutes, corresponding to three consecutive 5 min sampling intervals. In addition, to reflect thermal stress and equipment-condition effects, samples with a loading rate above 115% were also labeled as heavy-overload-related high-risk samples when accompanied by high winding temperature, high top-oil temperature, rapid temperature rise, cooling limitation, or high static baseline risk. Otherwise, the sample was labeled as non-heavy-overload.

As shown in Table 3, heavy-overload samples account for 8.02%, 9.25%, and 9.70% of the training, validation, and test sets, respectively. These results indicate that heavy-overload events are minority-class samples in all three subsets, which is consistent with the practical rarity of severe transformer overload events. To address this class imbalance, weighted binary cross-entropy was adopted in the C-DFRA module, and the class weights were calculated only from the training set.

Although the dataset contains 6700 samples and supports the case-study validation of the proposed framework under chronological train–validation–test splitting, it is still collected from a single power plant. Therefore, the generalization ability across different power plants, transformer types, and operating environments should be further validated using multi-source field datasets in future work.

**Figure 5:**
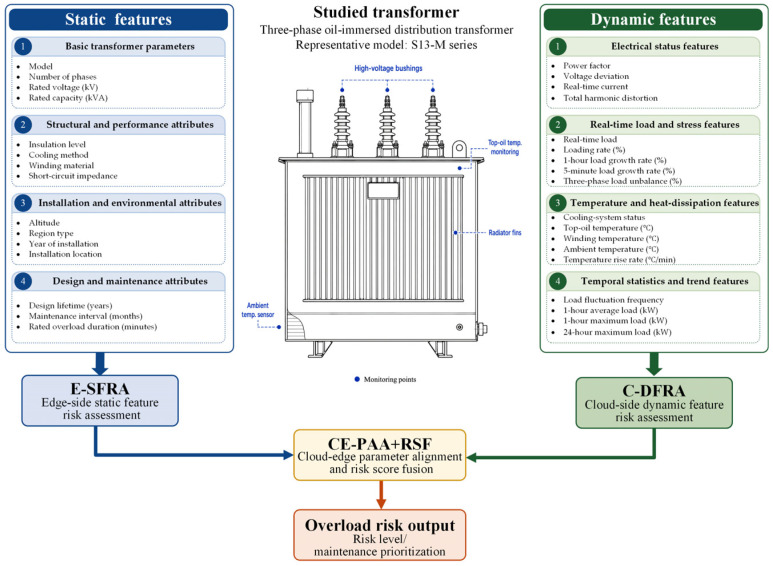
Schematic representation of the studied transformer and the used static/dynamic features.

To improve the clarity of the case-study description, a schematic representation of the studied transformer and the used input features is provided in Figure 5. Representative physical monitoring points related to top-oil temperature, ambient temperature, and the cooling structure are indicated around the transformer, while all static and dynamic features used in the model, including winding temperature and temporal load statistics, are summarized in the side panels. Static features are used by the E-SFRA module for edge-side static feature risk assessment, whereas dynamic features are used by the C-DFRA module for cloud-side dynamic feature risk assessment.

### 5.2. Validation Method

To comprehensively evaluate model performance, we employ the following metrics: KS Curve [34], cumulative gain curve [35], lift curve [34], Precision@K [36], calibration (reliability) curve and ECE [37], Brier score [38], log loss [38].

The evaluation metrics are described as follows:

1. KS Curve:

The KS curve, with threshold τ on the horizontal axis and KS(τ)=TPR(τ)−FPR(τ) on the vertical axis, is geometrically equivalent to the maximum vertical spacing between the cumulative distributions of the positive and negative sample scores (CDFs), reflecting the categorical separability. The maximum KS value is the key quantitative metric, where a larger value is better, and its corresponding threshold is often used as a practical decision point when needed.

2. Cumulative Gain Curve:

The samples are included progressively from high to low scores, with a cumulative gain curve giving the “proportion of positive cases captured in the first K per cent of coverage”, and a lift curve giving the multiplier of improvement relative to random selection. The key quantification point is the gain at a number of coverage rates; the higher up the curve to the left (Gain), the more positive cases are captured and the more efficient the strategy is for the same coverage cost.

3. Lift Curve:

At a given coverage, lift measures how many times better the model is than random selection—i.e., the ratio of precision within the top-α% to the overall positive rate. A higher lift (especially at small α) indicates the model brings true faults to the top earlier. This directly reflects operational value when only a small portion of assets can be inspected.

4. Precision@K:

Sort samples in descending order of predicted probability, compute the precision within the top K (or top K%), and plot it against K to measure screening efficiency under limited review resources. For a fixed audit budget, a higher and more slowly decaying curve indicates greater detection efficiency and stability.

5. Calibration (Reliability) Curve and ECE:

The reliability curve bins predicted probabilities and compares, for each bin, the average predicted probability with the observed frequency, assessing whether scores are trustworthy for threshold- or cost-sensitive decisions. The Expected Calibration Error (ECE) summarizes these discrepancies and a lower ECE indicates more reliable probabilities.

6. Brier Score:

The Brier score is the mean squared error between predicted probabilities and binary ground-truth labels, capturing both accuracy and calibration as a proper scoring rule. A lower value indicates higher probabilistic quality. It complements ranking metrics by emphasizing “being confidently correct” rather than merely ranking correctly.

7. Log Loss:

Log loss measures the consistency between predicted probabilities and actual outcomes, penalizing over-confident but wrong predictions more heavily. A lower value indicates better probabilistic consistency. Unlike threshold-dependent metrics, log loss is threshold-agnostic and more sensitive to both the sharpness of probabilities and their correctness.

### 5.3. Results and Analyses

To verify the effectiveness of the proposed TORA method, we compare it with several classifiers and fusion baselines. Specifically, UAG-NoUQ is used as an ablation variant by removing uncertainty quantification from TORA. To further examine whether the proposed RSF module is more effective than a simpler nonlinear fusion strategy, we introduce TORA-MLP-Fusion, in which the E-SFRA and C-DFRA branches are kept unchanged, while the final RSF module is replaced by a small multilayer perceptron. In addition, LightGBM [39], LSTM [40], and XGBoost [41] are used as representative baseline models for transformer overload prediction.

**Figure 6:**
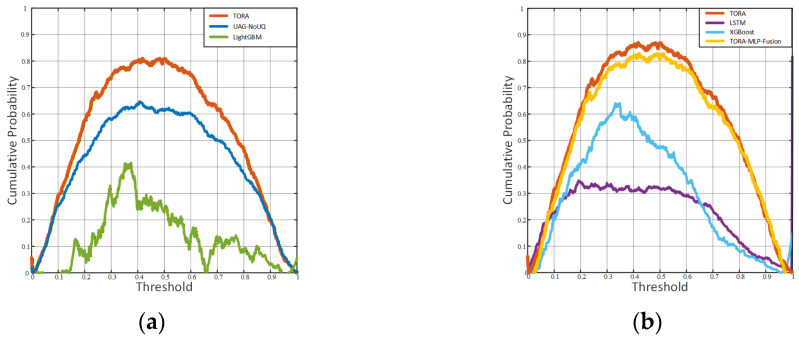
KS Curves (**a**) KS Curves of TORA, UAG-NoUQ and LightGBM; (**b**) KS Curves of TORA, LSTM, XGBoost and TORA-MLP-Fusion.

Figure 6 compares the KS curves of TORA, TORA-MLP-Fusion, UAG-NoUQ, LightGBM, LSTM, and XGBoost. The KS curve reflects the separability between heavy-overload and normal samples under different warning thresholds. As shown in Figure 6 and Table 4, TORA achieves the highest KS value and stays above the other models over a wide threshold range, indicating stronger risk discrimination and lower threshold sensitivity. From a physical perspective, this means that TORA can more consistently separate operating states with accumulated thermal-electrical stress from normal load fluctuations. Transformer heavy-overload risk is not determined by loading rate alone, but is related to the joint effect of equipment condition, load level, current, oil temperature, winding temperature, cooling capability, and temperature rise. In TORA, static features describe the long-term risk-bearing capability of the transformer, while dynamic features describe the short-term operating stress and thermal accumulation process. Their fusion through RSF makes the warning score more consistent with the actual overload formation process. TORA-MLP-Fusion remains competitive but is slightly lower than TORA, suggesting that a generic MLP can learn part of the nonlinear interaction, but it lacks the explicit uncertainty-aware fusion mechanism and engineering interpretability of RSF. Therefore, the higher and more stable KS curve of TORA provides more credible support for field warning, load transfer, cooling control, and maintenance prioritization.

**Figure 7:**
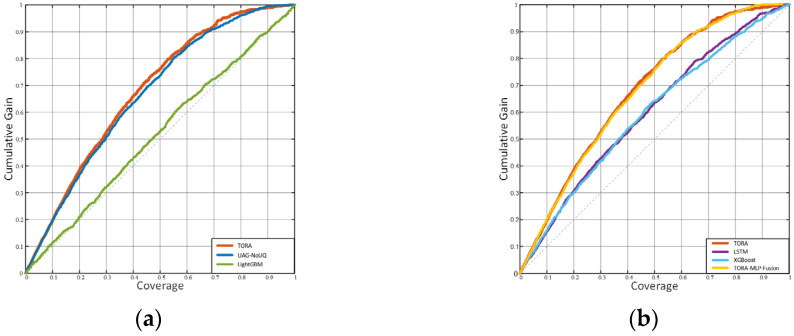
Cumulative gain curves: (**a**) cumulative gain curves of TORA, UAG-NoUQ and LightGBM; (**b**) cumulative gain curves of TORA, LSTM, XGBoost and TORA-MLP-Fusion.

Figure 7 compares the cumulative gain curves of TORA, TORA-MLP-Fusion, UAG-NoUQ, LightGBM, LSTM, and XGBoost. The cumulative gain curve reflects how many heavy-overload samples can be captured when only a certain proportion of top-ranked high-risk samples are inspected. As shown in Figure 7, TORA maintains a higher gain over most coverage ranges, especially in the O&M-relevant low-coverage region, indicating that it can identify more high-risk transformers under a limited inspection budget. This result is physically interpretable because heavy-overload risk is related to both inherent equipment vulnerability and real-time thermal-electrical stress accumulation. By integrating static risk-bearing information and dynamic operating stress through RSF, TORA can rank operating states with higher physical overload potential ahead of normal load fluctuations. TORA-MLP-Fusion also performs competitively but remains slightly lower than TORA, suggesting that a generic MLP can capture part of the nonlinear interaction but lacks the explicit uncertainty-aware fusion and engineering interpretability of RSF. Therefore, the higher cumulative gain of TORA means that field engineers can detect more potential overload risks with the same inspection budget, or achieve the same risk coverage with fewer inspections.

**Figure 8:**
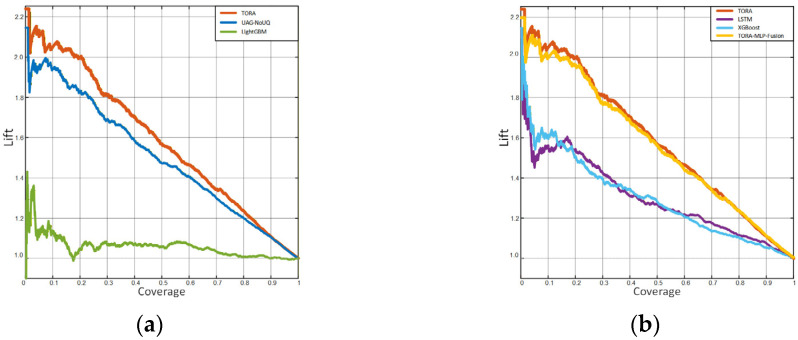
Lift curves: (**a**) lift curves of TORA, UAG-NoUQ and LightGBM; (**b**) lift curves of TORA, LSTM, XGBoost and TORA-MLP-Fusion.

Figure 8 compares the lift curves of TORA, TORA-MLP-Fusion, UAG-NoUQ, LightGBM, LSTM, and XGBoost. The lift curve measures the model’s ability to capture heavy-overload samples more efficiently than random inspection. As shown in Figure 8, TORA maintains higher lift values over most coverage ranges, especially in the low-coverage region, indicating stronger early risk capture capability. This result is physically interpretable because TORA ranks samples according to both static equipment vulnerability and dynamic thermal-electrical stress. These factors correspond to the actual formation process of transformer heavy overload, involving load level, current, oil temperature, winding temperature, cooling capability, temperature rise, and long-term equipment condition. Through RSF, TORA places physically riskier operating states earlier in the warning list. TORA-MLP-Fusion remains competitive but is slightly lower than TORA, showing that a generic MLP lacks the explicit uncertainty-aware fusion and engineering interpretability of RSF. Therefore, the higher lift curve of TORA can help engineers identify more true high-risk transformers in the first inspection batch and improve O&M efficiency.

**Figure 9:**
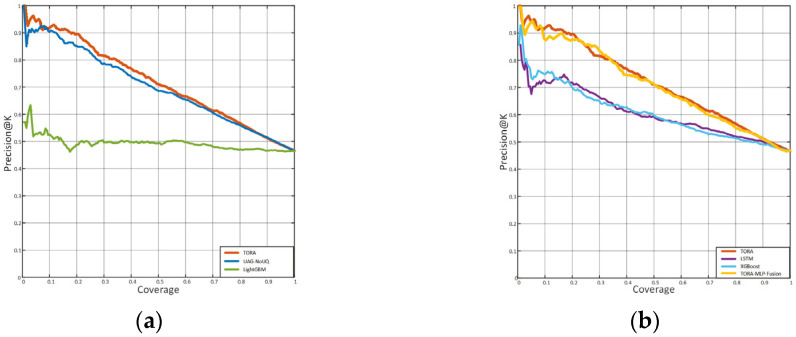
Precision@K Curves (**a**) Precision@K Curves of TORA, UAG-NoUQ and LightGBM; (**b**) Precision@K Curves of TORA, LSTM, XGBoost and TORA-MLP-Fusion.

Figure 9 compares Precision@K curves for TORA, UAG-NoUQ, LightGBM, LSTM, and XGBoost. TORA remains above all baselines across almost the entire coverage range and shows the flattest segment in the O&M critical region of low to medium coverage, indicating strong early ranking quality and robustness to threshold choice. UAG-NoUQ ranks second but decays more rapidly as coverage increases, while LSTM and XGBoost stay close to each other and clearly below TORA. LightGBM hovers only slightly above the base rate. Overall, static dynamic risk fusion with cloud calibration enhances fault prioritization and inspection efficiency for field crews under limited budgets.

**Figure 10:**
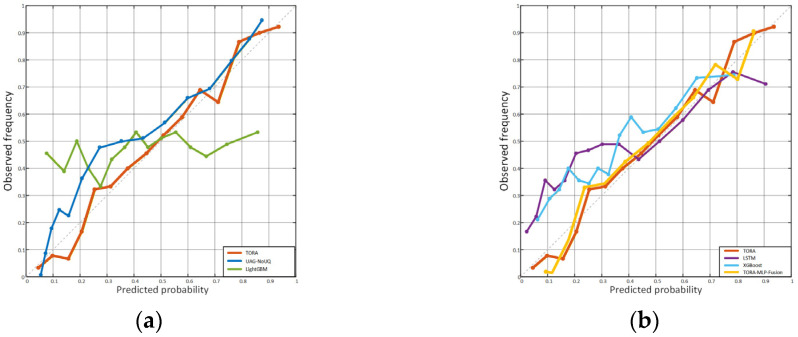
Calibration curves: (**a**) calibration curves of TORA, UAG-NoUQ and LightGBM; (**b**) calibration curves of TORA, LSTM, XGBoost and TORA-MLP-Fusion.

Figure 10 compares the calibration curves of TORA, TORA-MLP-Fusion, UAG-NoUQ, LightGBM, LSTM, and XGBoost. The calibration curve reflects whether the predicted overload probability matches the observed heavy-overload frequency. As shown in Figure 10 and Table 5, TORA is closer to the diagonal line, indicating reliable risk probability estimation. This result is physically meaningful because transformer heavy-overload risk is related to thermal-electrical stress accumulation rather than a binary classification result. By combining static equipment vulnerability, dynamic operating stress, and cloud-side calibration, TORA produces probabilities that better match overload occurrence. TORA-MLP-Fusion is competitive but slightly less stable than TORA, suggesting that a generic MLP lacks the explicit uncertainty-aware fusion and calibration mechanism of RSF. Therefore, TORA can provide more credible support for warning threshold setting, risk grading, and maintenance decisions.

Table 6 and Table 7 report the Brier score and NLL of all models, where lower values indicate better probability quality. TORA achieves the lowest Brier score and NLL, followed by TORA-MLP-Fusion and UAG-NoUQ, indicating that its predicted overload probabilities are closer to the observed outcomes and less affected by overconfident errors. This result is physically meaningful because transformer heavy-overload risk should be evaluated as a probabilistic process related to thermal-electrical stress accumulation, rather than only as a binary label. By combining static equipment vulnerability, dynamic operating stress, and cloud-side calibration, TORA produces sharper but still reliable risk probabilities. TORA-MLP-Fusion also improves probability estimation, but it remains slightly weaker than TORA, suggesting that a generic MLP lacks the explicit uncertainty-aware fusion and calibration mechanism of RSF. Therefore, TORA provides more credible probability support for warning threshold setting, risk grading, and cost-sensitive maintenance decisions.

To assess computational timeliness during online inference, we decompose a single inference into three stages—$T_{pre}$ (pre-processing), $T_{infer}$ (inference), and $T_{post}$ (post-processing)—and report their p50 and p95 (in ms). Here, $T_{pre}$ denotes the device-side online dynamic pre-processing time. For fairness, normalization and encoding of static features are treated as offline pipeline initialization and are excluded from $T_{pre}$. $T_{infer}$ is the model forward-pass time, i.e., the pure compute cost from the end of pre-processing to the production of raw scores/probabilities. $T_{post}$ is the post-processing time and excludes persistence and logging. The p50 (median) characterizes typical latency; the p95 (95th percentile) reflects tail jitter and worst-case service capacity. To avoid conflating communication effects with algorithmic differences, all three stages are reported as compute-only latencies, excluding network transfer, queuing, and scheduling overhead. All reported latency values are per-sample: we time each of the 6700 test samples to obtain a distribution for each stage and then report its p50/p95; therefore, these numbers are not the cumulative time for all 6700 samples.

**Figure 11:**
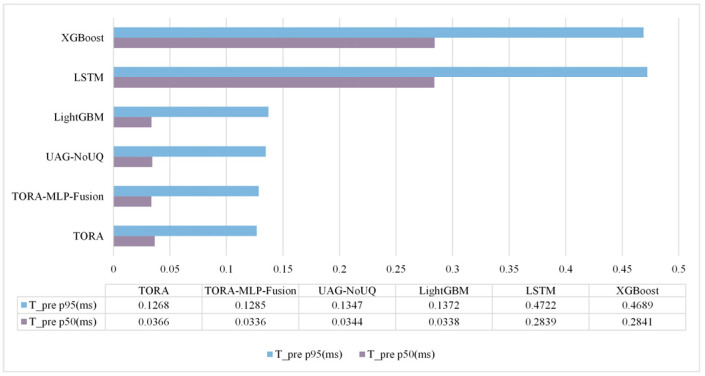
Comparison of model pre-processing time.

**Figure 12:**
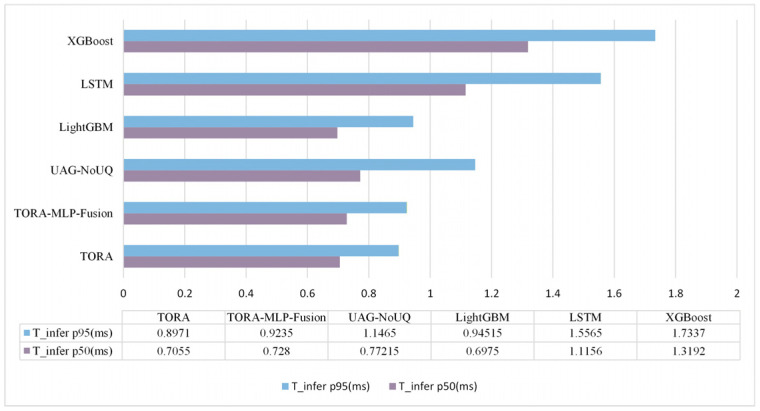
Comparison of model inference time.

**Figure 13:**
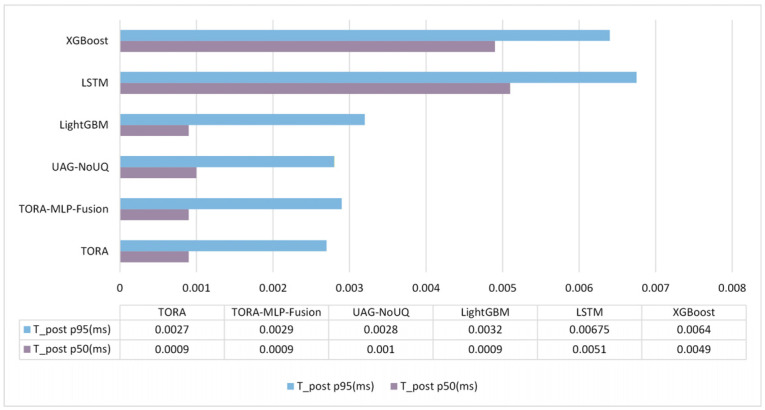
Comparison of model post-processing time.

The pre-processing, inference, and post-processing latencies are compared in Figure 11, Figure 12, and Figure 13, respectively.

In the pre-processing stage, TORA, TORA-MLP-Fusion, UAG-NoUQ, and LightGBM exhibit very low latency, clearly outperforming LSTM and XGBoost. This indicates that online dynamic pre-processing imposes negligible overhead for the proposed framework. TORA-MLP-Fusion shows a pre-processing latency close to TORA because it uses the same static and dynamic input pipeline. In the inference stage, LightGBM attains the fastest p50, while TORA remains highly competitive and shows stable tail latency. TORA-MLP-Fusion introduces only a slight additional inference cost compared with TORA, since it replaces the final RSF module with a small MLP while keeping the E-SFRA and C-DFRA branches unchanged. Compared with LSTM and XGBoost, TORA and TORA-MLP-Fusion still maintain much lower inference latency, indicating better suitability for online risk warning. In the post-processing stage, TORA and TORA-MLP-Fusion also maintain very low latency, and the differences among lightweight models are negligible. LSTM and XGBoost show slightly higher post-processing latency, but this stage has limited impact on the total online response time. Overall, the computational bottleneck mainly lies in the inference stage. Under the same measurement protocol, TORA achieves a good balance between physical interpretability, risk prediction performance, and online timeliness. The comparison with TORA-MLP-Fusion further shows that replacing RSF with a generic MLP only slightly increases latency but does not improve the overall risk assessment quality, supporting the efficiency and engineering suitability of the proposed RSF-based TORA framework.

### 5.4. Risk Distribution Prediction Case

**Figure 14:**
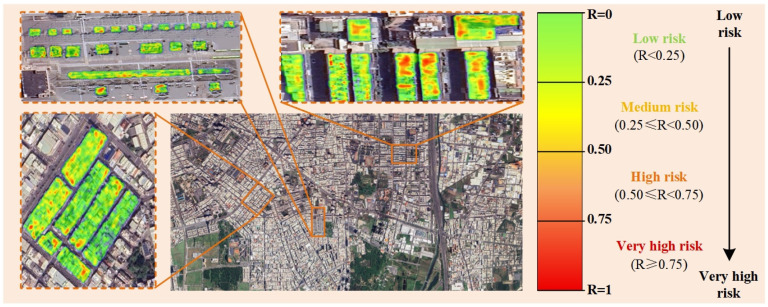
Regional transformer heavy-overload risk heatmap generated from calibrated TORA risk scores.

Based on the TORA model, we analyze the area surrounding a selected substation and jointly model the transformers’ static and dynamic features. Figure 14 presents a regional overload-risk heatmap generated from the calibrated TORA risk scores. Specifically, the static and dynamic features of each transformer are first input into the E-SFRA and C-DFRA modules, respectively. The resulting static and dynamic risk scores are then aligned and fused through the CE-PAA and RSF modules to obtain the calibrated overload-risk probability R for each transformer. The obtained risk probabilities are spatially associated with the corresponding transformer locations and overlaid on a real-world basemap to produce the regional risk distribution shown in Figure 14.

The risk levels in Figure 14 are defined according to the calibrated overload-risk probability R, which integrates both static and dynamic feature information. In this study, the probability interval [0,1] is divided into four response levels: low risk for R < 0.25, medium risk for 0.25 ≤ R < 0.50, high risk for 0.50 ≤ R < 0.75, and very high risk for R ≥ 0.75. Accordingly, the color scale increases monotonically from green to red with the overload-risk probability, where green, yellow, orange, and red correspond to low-, medium-, high-, and very high-risk levels, respectively. These thresholds are used for visual risk interpretation and maintenance prioritization.

For low-risk (green) areas, routine inspections are performed at the normal frequency. For medium-risk (yellow) areas, temperature verification or infrared thermography inspection is recommended within two weeks. For high-risk (orange) areas, a load-shedding or load-transfer plan should be formulated, cooling measures should be activated, and temperature/current tracking should be strengthened. For very-high-risk (red) areas, immediate load reduction or transfer is required, and outage inspection should be scheduled if continuous operation cannot be safely maintained.

## 6. Conclusions

To assist in more reasonable overhaul resource allocations in distributed renewable energy systems, this paper proposes a prediction method to diagnose future transformer overload spatiotemporal risks. From the perspective of sensor applications, the proposed framework converts heterogeneous monitoring observations into calibrated overload-risk information for practical transformer condition monitoring and maintenance support. The main works are outlined as follows:

1. Time-scale mismatch resolution: Modeling static (long-term drift) and dynamic (short-term fluctuation) features with E-SFRA and C-DFRA, respectively, to consider the distinct distributional properties of both two categories for less inherent time-scale mismatch.
2. Periodic calibration mechanism: The CE-PAA model is initiated for periodic parameter calibration, to form a robust system-wide feedback loop for better diagnosis performance.
3. Uncertainty-aware adaptive fusion: Under RSF ensemble, the static and dynamic risk scores are robustly combined via uncertainty-aware adaptive weighting in accordance with branch uncertainties, to bring about a cooperative integration of cloud–edge advantages.
4. Empirical evaluation and validation: A curve-based evaluation suite is conducted on the studied transformer dataset, and comparative analyses show that TORA achieves favorable performance in this case study. The results indicate its potential value for transformer condition monitoring, sensor data-based risk interpretation, and maintenance decision support.

It should also be noted that the empirical case in this study is based on monitoring records from a single power plant with 6700 samples. In addition, the heavy overload label definition includes expert informed thermal state conditions. Therefore, the current results mainly demonstrate the effectiveness of TORA in the studied case, while its cross scenario generalization ability across different power plants, transformer types, load profiles, and operating environments still requires further validation. Future work will incorporate multi source field datasets and cross site validation to further assess the transferability and robustness of the proposed framework.

## Figures and Tables

**Table 1:** Static features considered for transformer heavy-overload status assessment.

Categories of Static Features	Static Features
Basic Transformer Parameters	Model
	Number of phases
	Rated voltage (kV)
	Rated capacity (kVA)
Structural and Performance Attributes	Insulation level
	Cooling method
	Winding material
	Short-circuit impedance
Installation and Environmental Attributes	Year of installation
	Region type
	Altitude
	Installation location
Design and Maintenance Attributes	Rated overload duration (minutes)
	Maintenance interval (months)
	Design lifetime (years)

**Table 2:** Dynamic features considered for transformer heavy-overload status assessment.

Categories of Dynamic Features	Dynamic Features
Electrical status features	Power factor
	Voltage deviation
	Real-time current
	Total harmonic distortion
Real-time load and stress features	Real-time load
	Loading rate (%)
	1 h load growth rate (%)
	5 min load growth rate (%)
	Three-phase load unbalance (%)
Temperature and heat-dissipation features	Cooling-system status
	Top-oil temperature (°C)
	Winding temperature (°C)
	Ambient temperature (°C)
	Temperature rise rate (°C/min)
Temporal statistics and trend features	Load fluctuation frequency
	1 h average load (kW)
	1 h maximum load (kW)
	24 h maximum load (kW)

**Table 3:** Class distribution of the training, validation, and test sets.

Dataset	Number of Samples	Positive Samples	Negative Samples	Positive Ratio	Negative Ratio
Training set	5360	430	4930	8.02%	91.98%
Validation set	670	62	608	9.25%	90.75%
Test set	670	65	605	9.70%	90.30%

**Table 4:** KS values of TORA, UAG-NoUQ, LightGBM, LSTM, XGBoost and TORA-MLP-Fusion.

Models	KS Value
TORA	0.765
TORA-MLP-Fusion	0.735
UAG-NoUQ	0.662
LightGBM	0.203
LSTM	0.577
XGBoost	0.631

**Table 5:** Performance: ECE of TORA, UAG-NoUQ, LightGBM, LSTM, XGBoost and TORA-MLP-Fusion.

Models	ECE
TORA	0.036
TORA-MLP-Fusion	0.052
UAG-NoUQ	0.075
LightGBM	0.168
LSTM	0.134
XGBoost	0.117

**Table 6:** Performance: Brier scores of TORA, UAG-NoUQ, LightGBM, LSTM, XGBoost and TORA-MLP-Fusion.

Models	Brier Scores
TORA	0.1650
TORA-MLP-Fusion	0.1712
UAG-NoUQ	0.1766
LightGBM	0.2874
LSTM	0.2476
XGBoost	0.2416

**Table 7:** Performance: NLL scores of TORA, UAG-NoUQ, LightGBM, LSTM, XGBoost and TORA-MLP-Fusion.

Models	NLL
TORA	0.4976
TORA-MLP-Fusion	0.5148
UAG-NoUQ	0.5262
LightGBM	0.8057
LSTM	0.7253
XGBoost	0.6903

## Data Availability

The data presented in this study is available on request from the corresponding author. The data is not publicly available due to privacy or ethical restrictions.
